# Poly(2‐Hydroxyethyl Methacrylate) Hydrogel‐Based Microneedles for Metformin Release

**DOI:** 10.1002/gch2.202300002

**Published:** 2023-07-05

**Authors:** Manoj B. Sharma, Özlem Kap, Hend A. M. Abdelmohsen, Mark D. Ashton, Garry R. Harper, Melike Firlak, Jasmine E. Aaltonen, Kerry A. Bolland, Ryan Bragg, Sarah Deeley, Ella Francis, Nahin Kazi, Bethany L. Mapley, Vasileios Oikonomou, Amal D. Aljohani, David Cheneler, Volkan Kilic, Nesrin Horzum, John G. Hardy

**Affiliations:** ^1^ Department of Chemistry Lancaster University Lancaster LA1 4YB UK; ^2^ School of Engineering Lancaster University Lancaster LA1 4YW UK; ^3^ Department of Engineering Sciences Izmir Katip Celebi University Izmir 35620 Turkey; ^4^ Department of Pharmaceutics and Industrial Pharmacy Faculty of Pharmacy Ain Shams University African Union Organization Street Abbassia Cairo 11566 Egypt; ^5^ Department of Chemistry Gebze Technical University Gebze 41400 Turkey; ^6^ Department of Chemistry (Female Section) Faculty of Science King Abdulaziz University Jeddah‐Rabbigh 21589 Saudi Arabia; ^7^ Materials Science Institute Lancaster University Lancaster LA1 4YB UK; ^8^ Department of Electrical and Electronics Engineering Izmir Katip Celebi University Izmir 35620 Turkey

**Keywords:** aging, cancer, diabetes, drug delivery, hydrogels, microneedles

## Abstract

The release of metformin, a drug used in the treatment of cancer and diabetes, from poly(2‐hydroxyethyl methacrylate), pHEMA, hydrogel‐based microneedle patches is demonstrated in vitro. Tuning the composition of the pHEMA hydrogels enables preparation of robust microneedle patches with mechanical properties such that they would penetrate skin (insertion force of a single microneedle to be ≈40 N). Swelling experiments conducted at 20, 35, and 60 °C show temperature‐dependent degrees of swelling and diffusion kinetics. Drug release from the pHEMA hydrogel‐based microneedles is fitted to various models (e.g., zero order, first order, second order). Such pHEMA microneedles have potential application for transdermal delivery of metformin for the treatment of aging, cancer, diabetes, etc.

## Graphical abstract

Microneedle arrays are minimally invasive drug delivery devices that can be designed to deliver a variety of payloads the skin and other targets. Here, their application for the release of metformin in vitro is reported, which may combat aging, cancer, diabetes, etc.

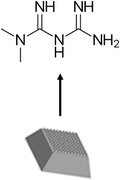

## 1 Introduction

Microneedle arrays are minimally invasive drug delivery devices that can be designed to deliver of a broad spectrum of payloads (e.g., drugs, vaccines, etc.).^[^ ^1^, ^2^, ^3^, ^4^, ^5^, ^6^, ^7^, ^8^, ^9^ ^]^ The dimensions and structures of microneedle arrays are important factors in their delivery profiles and minimization of pain associated with their insertion.^[^ ^10^, ^11^ ^]^ While conventional transdermal patches are used for sustained drug delivery (often over several days), microneedle arrays tend to be employed for delivery of doses over relatively short periods of time.^[^ ^12^, ^13^, ^14^ ^]^ Drug release kinetics from microneedle arrays is determined by a variety of factors, including microneedle architecture, the material used to prepare the microneedle arrays, the method by which the drug/bioactive is incorporated in/on the microneedle array.^[^ ^13^, ^15^, ^16^, ^17^ ^]^ Microneedle arrays that respond to stimuli (e.g., electricity,^[^ ^8^, ^18^, ^19^ ^]^ light,^[^ ^20^ ^]^ sound,^[^ ^21^, ^22^ ^]^ others, and potentially combinations thereof)^[^ ^23^, ^24^, ^25^ ^]^ are an emerging class of arrays with exciting prospects for theranostic applications,^[^ ^26^, ^27^, ^28^, ^29^, ^30^, ^31^, ^32^ ^]^ and inclusion in wearable devices useful for a variety of healthcare applications,^[^ ^33^ ^]^ that have long term prospects for clinical impact,^[^ ^34^, ^35^ ^]^ particularly for the treatment of conditions where the use of needles is problematic (e.g., diabetes management).^[^ ^36^ ^,^ ^37^ ^]^

Microneedles are the subject of significant research and development, investigating different microneedle designs for drug delivery to the skin and other targets.^[^ ^38^ ^]^ Microneedles have been produced using a variety of different materials (including inorganic‐based materials (ceramics, metals, silicon, etc.),^[^ ^39^ ^]^ and carbon‐based materials (e.g., natural and synthetic polymers)) offering a rich palette of opportunities to optimize the delivery of their payloads for specific applications, with exciting examples of microneedle technologies for a variety of clinically/industrially relevant applications.^[^ ^6^, ^13^, ^40^, ^41^, ^42^, ^43^ ^]^

Polymer hydrogel‐based materials are popular for biomedical technologies owing to their breadth of properties due to their tunable chemistry, mechanics, structures, etc. which facilitates their application in drug delivery, tissue engineering, etc.^[^ ^19^, ^44^, ^45^, ^46^, ^47^, ^48^ ^]^ Of particular interest for microneedle‐based technologies is their relatively low cost and ease of disposal as medical waste in accordance with a country's legal regulations (e.g., incineration, sterilization, etc.).^[^ ^5^ ^]^ Metformin is a drug that is used in the treatment of diabetes^[^ ^49^, ^50^, ^51^, ^52^ ^]^ and cancer,^[^ ^53^, ^54^, ^55^, ^56^ ^]^ with emerging evidence of its potential for application in treating COVID‐19^[^ ^57^, ^58^ ^]^ and aging‐related conditions.^[^ ^59^, ^60^, ^61^, ^62^ ^]^

Here, we describe the application of additive manufacturing approach to prototype master casts for microneedle patches to facilitate the development of pHEMA hydrogel‐based microneedles. This method enabled the preparation of robust microneedle patches with mechanical properties such that they would penetrate skin, and the behavior of the pHEMA gels was studied with various practical/theoretical approaches to explain their swelling and payload release profiles.^[^ ^63^ ^]^

## 2 Results and Discussion

### 2.1 Microneedle Design, Production, and Characterization

**Figure 1:**
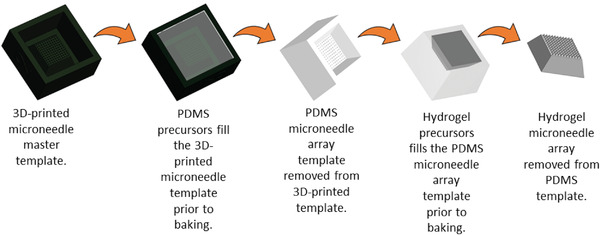
Hydrogel microneedle array production process.

Microneedle array master templates were designed via computer aided design (CAD), the technical drawings for microneedle templates with nine different geometries are depicted in Table S1 and Figures S1–S9 (Supporting Information). The 10×10 array master templates (composed of light curing methacrylic/acrylic resin, Envision TECH HTM 140 V2) were fabricated using 3D stereolithography (a low‐cost alternative to conventional microfabrication or laser micromachining, with a resolution of ≈15 µm). The master templates were sprayed with release liner, and filled with a polydimethylsiloxane (PDMS) precursor mixture (a SYLGARD 184 PDMS kit), which was degassed to diminish defects, and baked to crosslink the PDMS. After baking, the samples were cooled to room temperature, filled with various mixtures of hydrogel precursors (Table S2, Supporting Information) and baked to produce crosslinked pHEMA hydrogel‐based microneedle arrays. After baking, the samples were cooled to room temperature, and the dry pHEMA microneedle arrays were removed from the PDMS templates, swollen in water, and extensively washed to remove any contaminants (initiators, oligomers, species not covalently attached to the pHEMA hydrogel matrices, etc.). The microneedle array production process is summarized in **Figure** **1**, and a photograph of exemplars of the master template, PDMS template and pHEMA hydrogel‐based microneedle arrays in Figure S10 (Supporting Information).

Of the nine different microneedle array designs, we observed the most reliable microneedle array production from the PDMS microneedle array template with triangle/pyramid structures (design 9, Tables S1 and S2, and Figure S11, Supporting Information) and used that in all further studies (employing pHEMA hydrogels derived from baking hydrogel precursors (2‐hydroxyethyl methacrylate (HEMA), poly(ethylene glycol)dimethacrylate (PEGDMA, average Mn 550), and benzoyl peroxide (BPO)). The designed length of the microneedles in the 3D‐printed master mold for the triangle/pyramid microneedle arrays was 590 µm, and confocal microscopy showed the actual length of the triangle/pyramid pits in the PDMS templates was ≈316 ± 38 µm, and confocal showed the length of the pHEMA hydrogel microneedles produced was ≈238 ± 97 µm; deviations in desired/actual dimensions are the norm with this method of microneedle manufacture (further dimensional information are displayed in Table S3, Supporting Information).

Fourier transform infrared (FTIR) spectroscopy in attenuated total reflection (ATR) mode confirmed the successful polymerization of the monomer HEMA and cross‐linker PEGDMA initiated by BPO (Figure S12, Supporting Information).^[^ ^64^ ^]^ The FTIR spectrum showed a characteristically broad peak at ≈3400 cm^−1^ from O—H stretching, the peak at 2964 cm^−1^ is due to C—H stretching, the peak at 1719 cm^−1^ is characteristic of the ester group in pHEMA, the peak 1450 cm^−1^ is characteristic of C—H deformations, the peak at 1253 cm^−1^ is characteristic of O—H bending, the peak at 1148 cm^−1^ is characteristic of C—O stretching, the peak at 1071 cm^−1^ is characteristic of the ether in the cross‐linker, the peak at 720 cm^−1^ is characteristic of CH_2_ rocking (and the C=C stretch characteristic of non‐cross‐linked HEMA/PEGDMA (at ≈1637 cm^−1^ is not observed).

**Figure 2:**
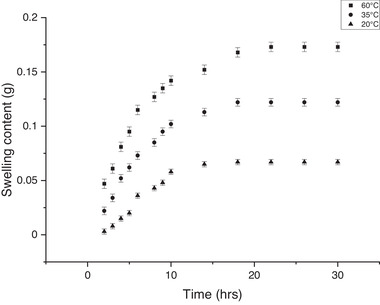
Hydrogel swelling at different times and temperatures.

Hydrogel swelling is controlled by the structure and dynamics of the constituent polymers and offers an insight into important properties including mechanics, water diffusion within the gels, and their capacity to be loaded with drugs.^[^ ^65^, ^66^ ^]^ Swelling studies were carried out at 20, 35, and 60 °C, observing that the swelling reached equilibrium after ≈24 h; with an initial rapid increase in gel mass (≈0–6 h), followed by slower swelling (≈6–24 h), until equilibrium; swelling was faster at higher temperatures because of the increase in thermal mobility of solvent and polymer chains (**Figure** **2** and Figure S13, Supporting Information). The data collected enabled calculation of swelling coefficients and swelling kinetics (non‐Fickian at 20 °C (anomalous diffusion due to crystallization, interactions between diffusant and polymers, molecular relaxation, segmental mobility retardation, etc.), and Fickian at 35 and 60 °C (controlled by thermodynamic forces by gradients of chemical potential and/or differences of concentrations); **Table** **1**; Table S4 and Figures S13–S16, Supporting Information) useful in understanding transport processes within polymer hydrogel matrices for medical and technical applications.^[^ ^67^ ^]^

**Table 1:** Parameters of swelling coefficients of the microneedle arrays and swelling kinetics

Temperature [°C]	*R* ^2^	*n* [m^2^ s^−1^]	*k* × 10^2^	D × 10^7^	Swelling kinetics
20	0.92	0.58	1.08 ± 1.10	0.80 ± 0.07	Non‐Fickian
35	0.94	0.46	2.94 ± 1.32	1.40 ± 0.11	Fickian
60	0.95	0.40	4.94 ± 2.41	1.90 ± 0.21	Fickian

### 2.2 In Vitro Validation of Microneedle Arrays

#### 2.2.1 Microneedle Mechanics

**Figure 3:**
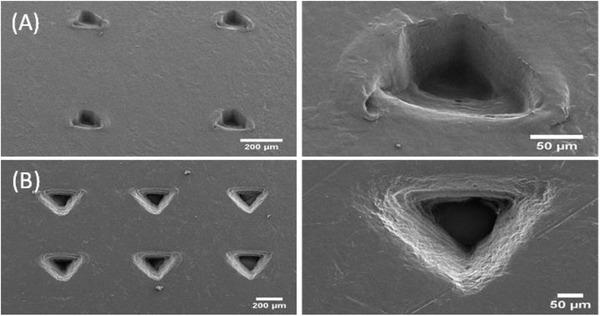
SEM micrographs of Parafilm multilayers after microneedles are inserted. A) Microneedles inserted with a force of 10 N. B) Microneedles inserted with a force of 40 N. Left hand scale bars represent 200 µm, right hand scale bars represent 50 µm.

Skin is a complex layered tissue: the protective surface layer is known as the epidermis (itself a multilayer tissue composed of the stratum corneum, stratum lucideum, stratum granulosum, stratum spinosum, and stratum basale) containing cells responsible for protection of the immune system and pigmentation; the dermis contains hair follicles, nerve endings, and oil/sweat glands; and the subcutaneous tissue is beneath, containing connective tissue, fat, larger blood vessels, etc. The thicknesses of each layer are variable depending on the location on the body (thicker on the soles of the feet than the eyelids) and person. Microneedles are typically designed to have lengths of ≈150–1500 µm to facilitate use without damaging the patient's nerves.^[^ ^3^, ^68^ ^]^ To mimic the mechanical properties of skin, a multilayer film composed of 8 layers of Parafilm was prepared, previously used to mimic microneedle array penetration into skin.^[^ ^69^ ^]^ An insertion force of 40 N was more effective than an insertion force of 10 N at penetrating the Parafilm (**Figure** **3**; Figure S17, Supporting Information). The length of the microneedles in this study was ≈238 ± 97 µm, which was reduced to ≈206 ± 108 µm after penetration into Parafilm, using an insertion force of 40 N (Figure S18, Supporting Information, N.B. differences in lengths are not statistically significant).

#### 2.2.2 Drug Delivery

**Figure 4:**
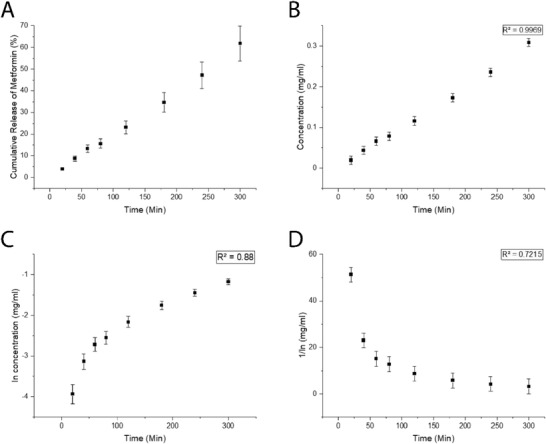
Metformin release study. a) Cumulative drug release. b) Drug release over 5 h fitted to zero order kinetics. c) Drug release over 5 h fitted to first order kinetics. d) Drug release over 5 h fitted to second order kinetics.

Metformin was loaded into the microneedle arrays before attaching a coin to the base of the microneedle arrays using Parafilm, this served to ensure the arrays sink into the phosphate buffered saline (PBS) release medium at pH 7.4 chosen to mimic the pH of blood into which the microneedles would release the drugs if applied transdermally.^[^ ^70^, ^71^, ^72^ ^]^ The average water content in the pHEMA microneedle arrays from swelling and dry weight analysis studies was observed to be ≈0.140 mL, and the microneedles were loaded in a solution of metformin in PBS (at the solubility limit of metformin in PBS, i.e., 1 g of metformin in 10 mL of PBS), consequently the drug loading capacity of the microneedle arrays is 14 mg of metformin, and an encapsulation efficiency of 98% ± 1% after two rounds of loading. The release of metformin release in vitro was assessed via UV–vis spectroscopy (the limit of detection (LoD) and limit of quantification (LoQ) were found to be 36.8 and 111.4 ppm, respectively), observing ≈60% of the payload to be released over the period of 300 min and quantitative release over the period of 24 h (**Figure** **4**a). The drug release profile data for the first 300 min were analyzed with different empirical release kinetics models (zero order, first order, second order) using the regression coefficient method, where the model fits the release mechanism if the regression coefficient value (*R* ^2^) is equal or close to 1. The slope of the Zero order release plot (Figure 4b) is linear with a regression coefficient *R* ^2^ = 0.99, by contrast the release of metformin does not display first or second order release kinetics as the regression coefficients (*R* ^2^) are lower and the graphs are not linear (Figure 4c,d, respectively).

## 3 Conclusion

The pHEMA hydrogel microneedle arrays described herein are inexpensive, simple to prepare and to load with drugs as demonstrated with metformin which is investigated for a variety of applications due to its interesting biological activity, including treatment of aging, cancer, and diabetes. The microneedles have mechanical properties suitable for penetrating skin (as demonstrated using a simple Parafilm model), and they can deliver therapeutically relevant quantities of metformin over a short space of time.^[^ ^74^ ^]^

The tunable nature of the microneedles (i.e., materials from which they are produced, microneedle morphology, etc.) offer opportunities to control the release kinetics, and the integration of such microneedle arrays within devices capable of sensing analytes^[^ ^75^, ^76^, ^77^, ^78^ ^]^ that are biomarkers of medical conditions and triggering the delivery of the drug from the microneedle arrays,^[^ ^18^, ^30^, ^71^, ^79^, ^80^, ^81^ ^]^ offers long term potential for controlling the delivery of the drug in line with the chronobiology of the condition being treated that offers significant potential savings to healthcare systems worldwide. Such microneedles are one of a host of potential methods of non‐invasive delivery technologies^[^ ^82^, ^83^ ^]^ being developed by multidisciplinary research and development teams of people based in academic, industrial, and clinical settings will be involved to translate unique technical know‐how to the benefit of humanity,^[^ ^6^ ^]^ and the interest in the application of microneedles for biomedical applications is demonstrated both by the volume of academic literature produced,^[^ ^12^, ^84^ ^]^ and moreover the fact that there are currently >500 entries including the term microneedles in their title, abstract or keyword in the Cochrane Central Register of Controlled Trials.

## 4 Experimental Section

### Materials

Unless otherwise noted, everything was purchased from Sigma–Aldrich (Gillingham, UK) and used as supplied.

### Preparation of PDMS Microneedle Templates

Microneedle array templates were produced via 3D printing with different microneedle structures (Table S1, Supporting Information). These templates were used to create microneedle molds using a SYLGARD 184 PDMS kit. A 10:1 mixture of silicone elastomer to silicon elastomer curing agent was stirred in a plastic container and degassed in a vacuum desiccator until bubbles stopped rising to the surface. The 3D printed templates were placed on a ceramic tile, sprayed with release liner (Ambersil Silicone Mould Release Agent Plastic, RS Components UK, Corby, UK), and allowed to dry. The templates were filled with the PDMS mixture (≈3 mL of the PDMS mixture was needed to fill them), baked in an oven at 60 °C for 16 h, after which they were cooled, removed from the templates using spatulas, and stored in plastic containers until use.

### Preparation of pHEMA Hydrogel‐Based Microneedles

Twenty milliliters of 2‐hydroxyethyl methacrylate (HEMA), 0.2 mL of PEGDMA (average Mn 550), and 88 mg of BPO were mixed until homogeneous, then degassed in a vacuum desiccator, followed by transfer of ≈3 mL of the formulation to the microneedle mold. The samples were heated at 100 °C in an oven for 3 h, after which they were cooled to room temperature and thoroughly washed with deionized water over a period of a week to remove any non‐crosslinked components (e.g., initiators, monomers, oligomers, etc.).

### Fourier Transform Infrared (FTIR) Spectroscopy

FTIR spectra were recorded in ATR mode using an Agilent Cary 630 FTIR spectrophotometer at room temperature between 700 and 4000 cm^−1^. Data representative of measurements carried out in triplicate are presented.

### Microneedle Insertion Study

The insertion of microneedle arrays into multilayer films of Parafilm (prepared by folding the Parafilm to obtain an 8‐layer film with a thickness of ≈1 mm thickness, used due to its comparable mechanics to skin), was studied as described previously in the literature,^[^ ^12^ ^]^ with minor adaptations described in the supporting information. All the experiments were in triplicate (*n* = 3), and data are reported as the mean average ± standard deviation. Microscopy was used to analyze the height of the microneedles before and after penetration into the multilayer films of Parafilm; analyzed using the software supplied with the digital microscope.

### Hydrogel Swelling

Swelling studies were carried out as described in the supporting information. All the experiments were in triplicate (*n* = 3), and data were reported as the mean average ± standard deviation. See Figures S12–S15 (Supporting Information), Table 1, and Table S3 (Supporting Information).

### Scanning Electron Microscopy (SEM)

Samples were coated with a thin layer of gold (≈10 nm) using a sputter coater prior to analysis with a Q150 RES. The morphology of the microneedle templates and indented Parafilm surfaces were analyzed by an SEM (Carl Zeiss 300VP, Germany)

### Confocal Microscopy

Geometry of microneedles and molds were measured using a LEXT OLS5000 3D measuring laser microscope. Images were taken with the MPLFLN10x LEXT objective lens with a 1× zoom in 3D standard and color mode. Images were 1024×1024 pixels in size equivalent to an area 1280 ± 1 µm by 1280 ± 1 µm. A single scan provided the geometry of a 2 × 2 array of microneedles. Larger images were created by stitching scans together. Postprocessing of images was conducted to remove point noise and inclination. Height data measured using two‐point analysis (tip of microneedles and base) taken from line data of 3D profile of microneedles. All measurements were at least in triplicate (*n* ≥ 3), and data were reported as the mean average ± standard deviation.

### Drug Delivery Studies

The maximum solubility of metformin in water is 1 g in 10 mL; the average water content in hydrogel microneedles from swelling/dry weight analysis is 0.140 mL, therefore the maximum amount of metformin in the microneedle arrays is 14 mg. Microneedle arrays were incubated in an excess volume of metformin solution (0.1 g mL^−1^) for 24 h at room temperature (4 patches in 20 mL of metformin solution), after which the microneedles transferred to fresh metformin solution for another 24 h to ensure high loading efficiency (98% ± 1% after 2 rounds of loading).

UV–vis spectra of samples were recorded using an Agilent Cary 60 UV–vis spectrophotometer (metformin *λ*_max_ at 625 nm) at various times and correlated to a calibration curve to enable assessment of cumulative release of metformin at 32 °C into PBS at pH 7.4 from coin‐weighted samples.^[^ ^70^ ^]^ A ten pence coin was attached to the back of Parafilm‐backed metformin‐loaded microneedle arrays with Parafilm, and placed inside a beaker containing 30 mL of PBS containing a stirrer at 100 strokes min^−1^. Samples from the PBS release medium (3 mL) were extracted at defined time intervals and replaced with an equal volume of PBS).^[^ ^70^ ^]^ Drug release kinetics and mechanism were assessed using standard literature methods^[^ ^1^ ^]^ described in full in the Supporting Information. All the experiments were in triplicate (*n* = 3), and data are reported as the mean average ± standard deviation.

## Conflict of Interest

The authors declare no conflict of interest.

## Author Contributions

J.E.A., K.A.B., R.B., S.D., E.F., N.K., and B.L.M. contributed equally to this work. The manuscript was written through contributions of all authors. All authors have given approval to the final version of the manuscript.

## Supporting information

Supporting Information

Supporting Information

## Data Availability

The data that support the findings of this study are available from the corresponding author upon reasonable request.
