# Supplementary material for: Poly(2‐Hydroxyethyl Methacrylate) Hydrogel‐Based Microneedles for Metformin Release
Source: Glob Chall. 2023 Jul 5;7(8):2300002. doi: 10.1002/gch2.202300002 (PMC10448145; doi:10.1002/gch2.202300002)
Supplement: Supplementary file 1 — Supporting Information [file GCH2-7-2300002-s002.pdf]

Global Challenges
Open Access

Supporting Information

for *Global Challenges.*, DOI 10.1002/gch2.202300002

Poly(2-Hydroxyethyl Methacrylate) Hydrogel-Based Microneedles for Metformin Release

Manoj B. Sharma, Özlem Kap, Hend A. M. Abdelmohsen, Mark D. Ashton, Garry R. Harper, Melike Firlak, Jasmine E. Aaltonen, Kerry A. Bolland, Ryan Bragg, Sarah Deeley, Ella Francis, Nahin Kazi, Bethany L. Mapley, Vasileios Oikonomou, Amal D. Aljohani, David Cheneler, Volkan Kilic*, Nesrin Horzum* and John G. Hardy**

Supporting Information

Poly(2-hydroxyethyl methacrylate) hydrogel-based microneedles for metformin release

Manoj B. Sharma, Özlem Kap, Hend A. M. Abdelmohsen, Mark D. Ashton, Garry R. Harper, Melike Firlak, Jasmine E. Aaltonen, Kerry A. Bolland, Ryan Bragg, Sarah Deeley, Ella Francis, Nahin Kazi, Bethany L. Mapley, Vasileios Oikonomou, Amal D. Aljohani, David Cheneler, Volkan Kilic,* Nesrin Horzum,* and John G. Hardy**

Table S1. Microneedle templates and their corresponding needle structure design.

Microneedle Array Design ID	Microneedle array design description	Figure with technical drawings
1	Cylinder based cone 1	S1
2	Cylinder based cone 2	S2
3	Pentagon based cone	S3
4	Pentagon pyramid	S4
5	Rocket needle	S5
6	Square based cone 1	S6
7	Square based cone 2	S7
8	Square pyramid	S8
9	Triangle pyramid	S9

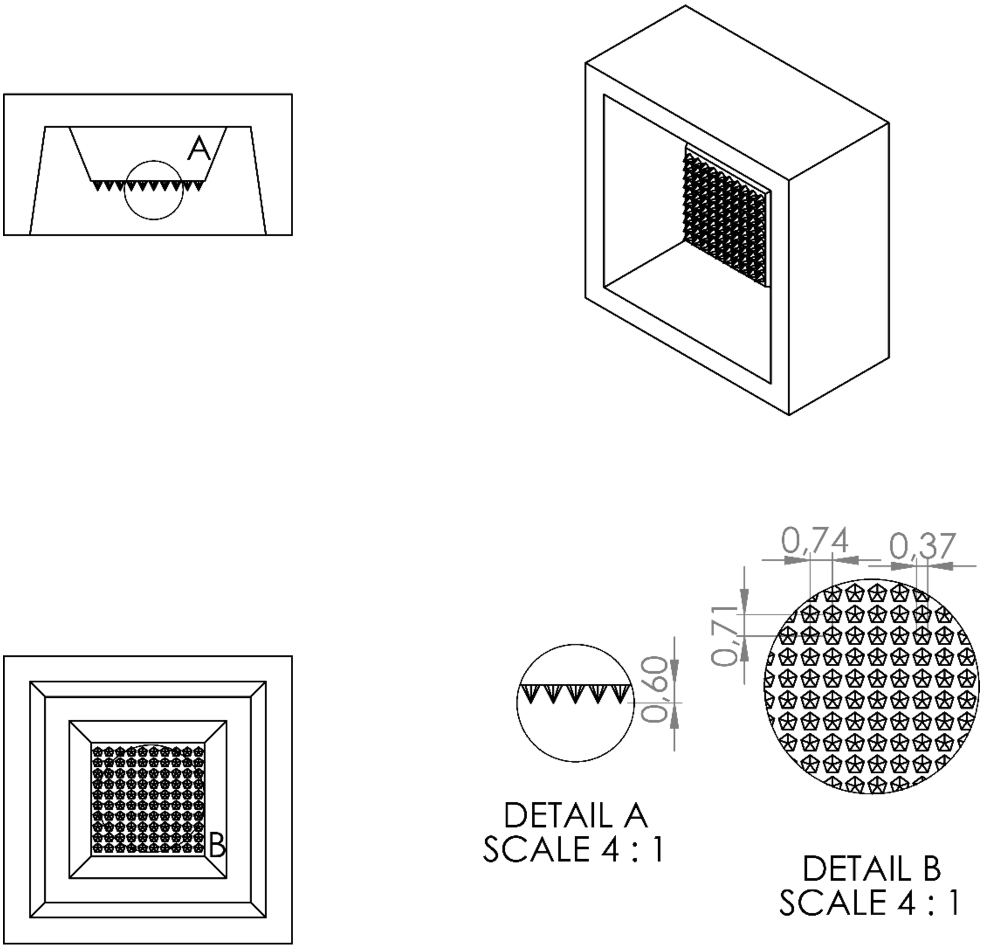

Figure S1. Technical drawings of 3D printed templates (Template to produce array design 1).

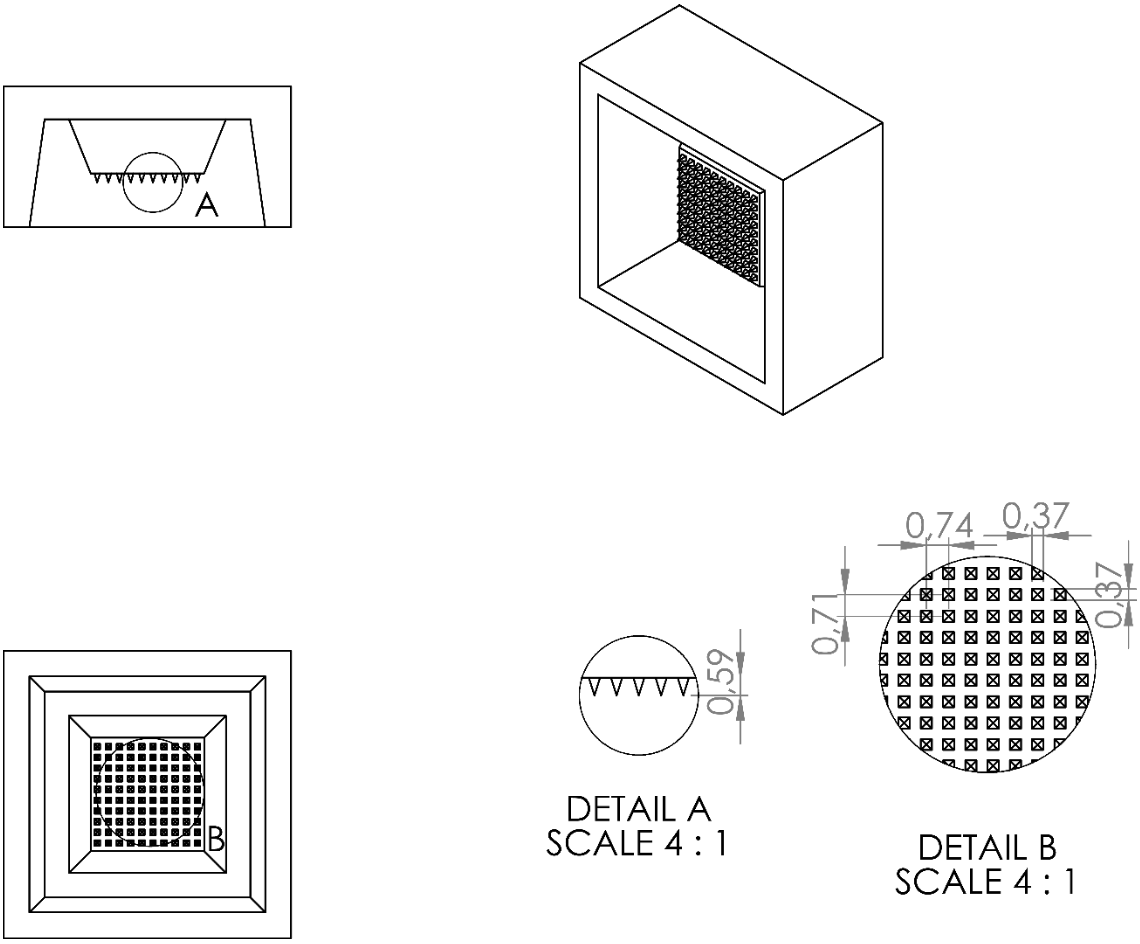

Figure S2. Technical drawings of 3D printed templates (Template to produce array design 2).

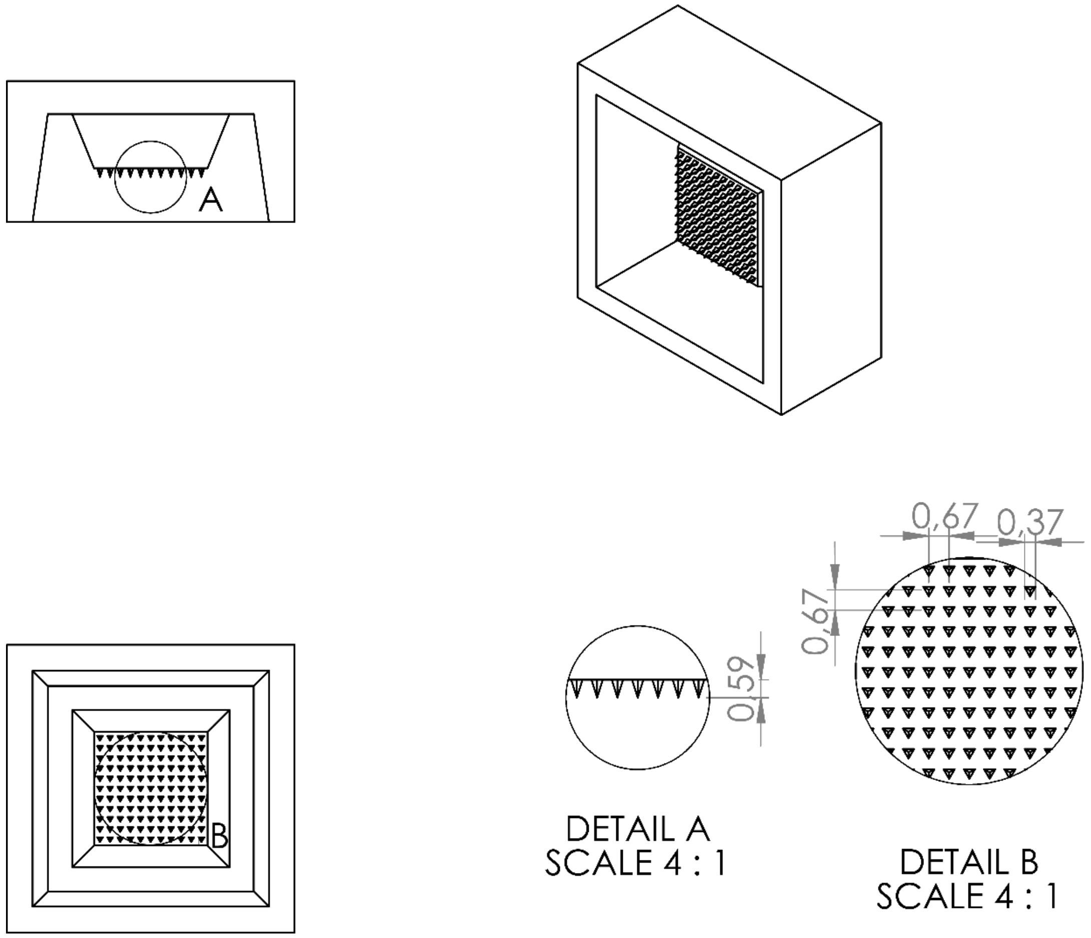

Figure S3. Technical drawings of 3D printed templates (Template to produce array design 3).

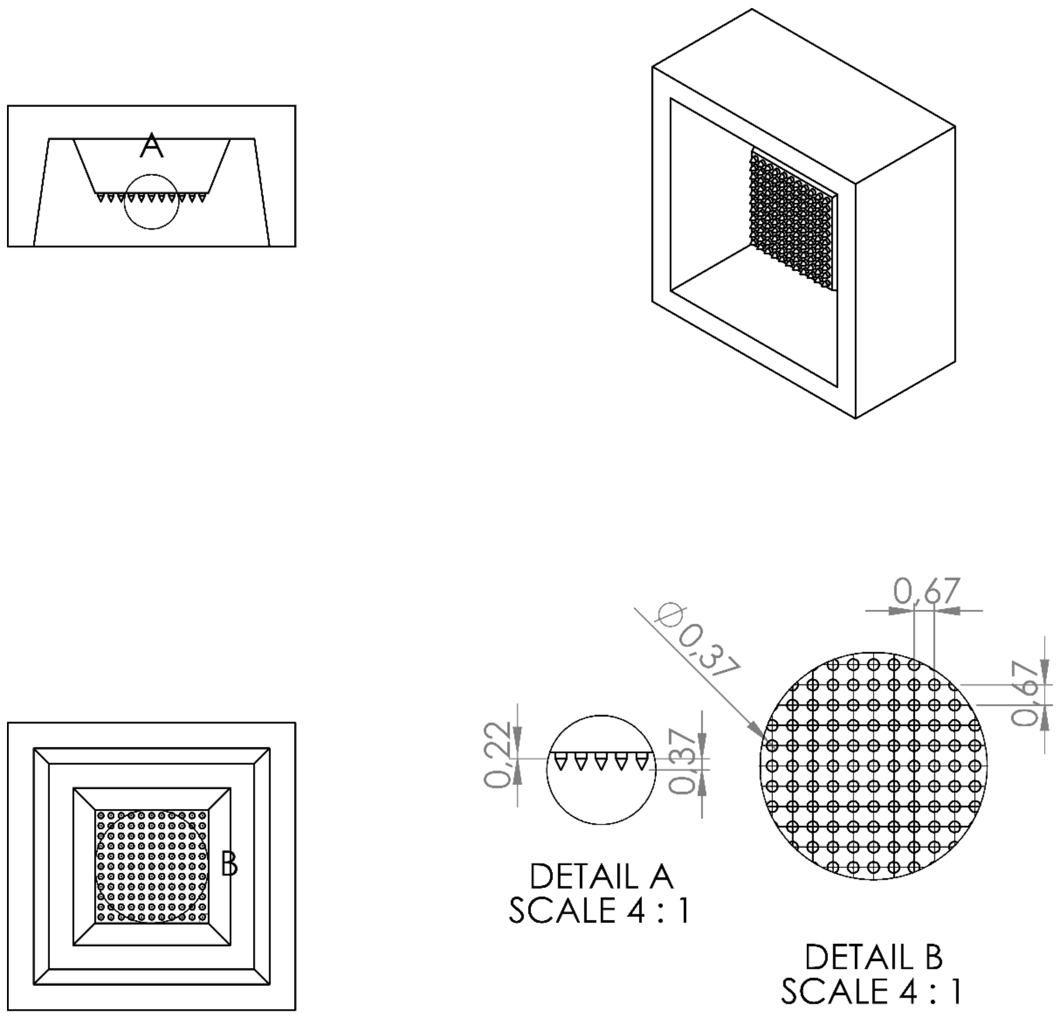

Figure S4. Technical drawings of 3D printed templates (Template to produce array design 4).

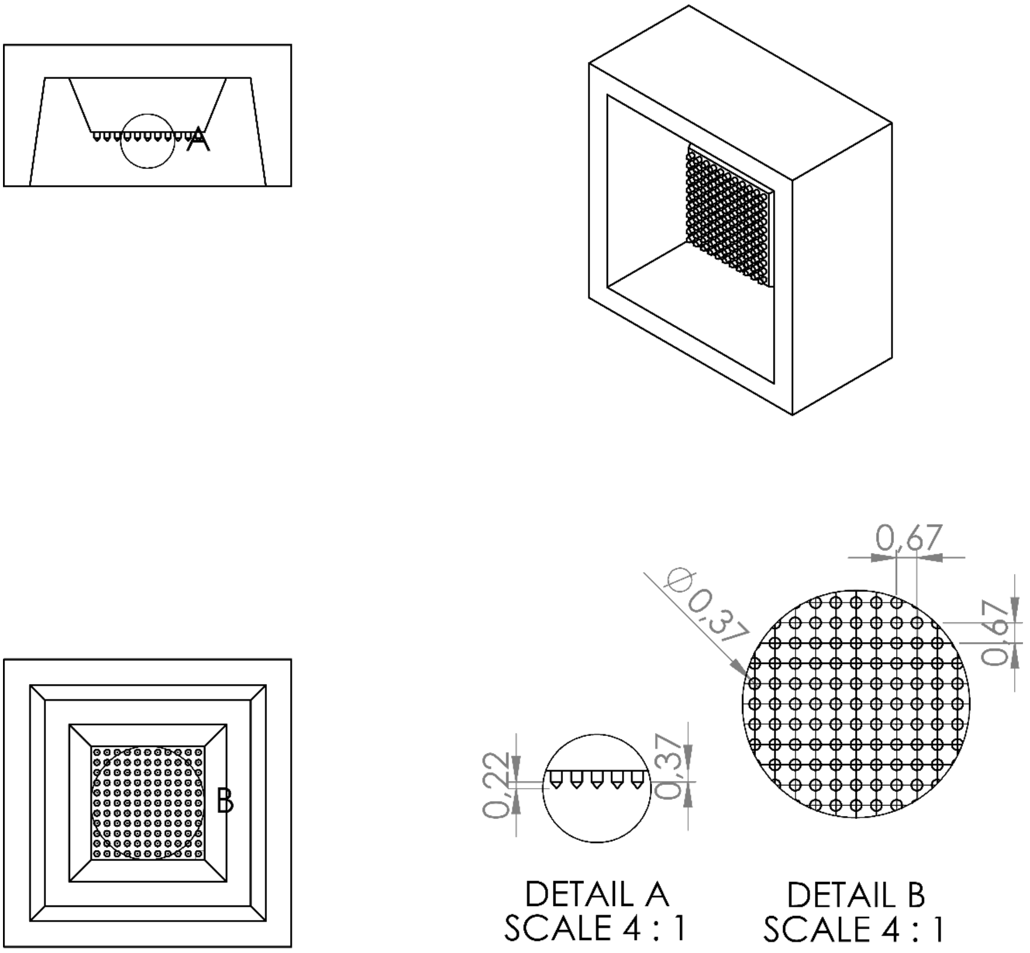

Figure S5. Technical drawings of 3D printed templates (Template to produce array design 5).

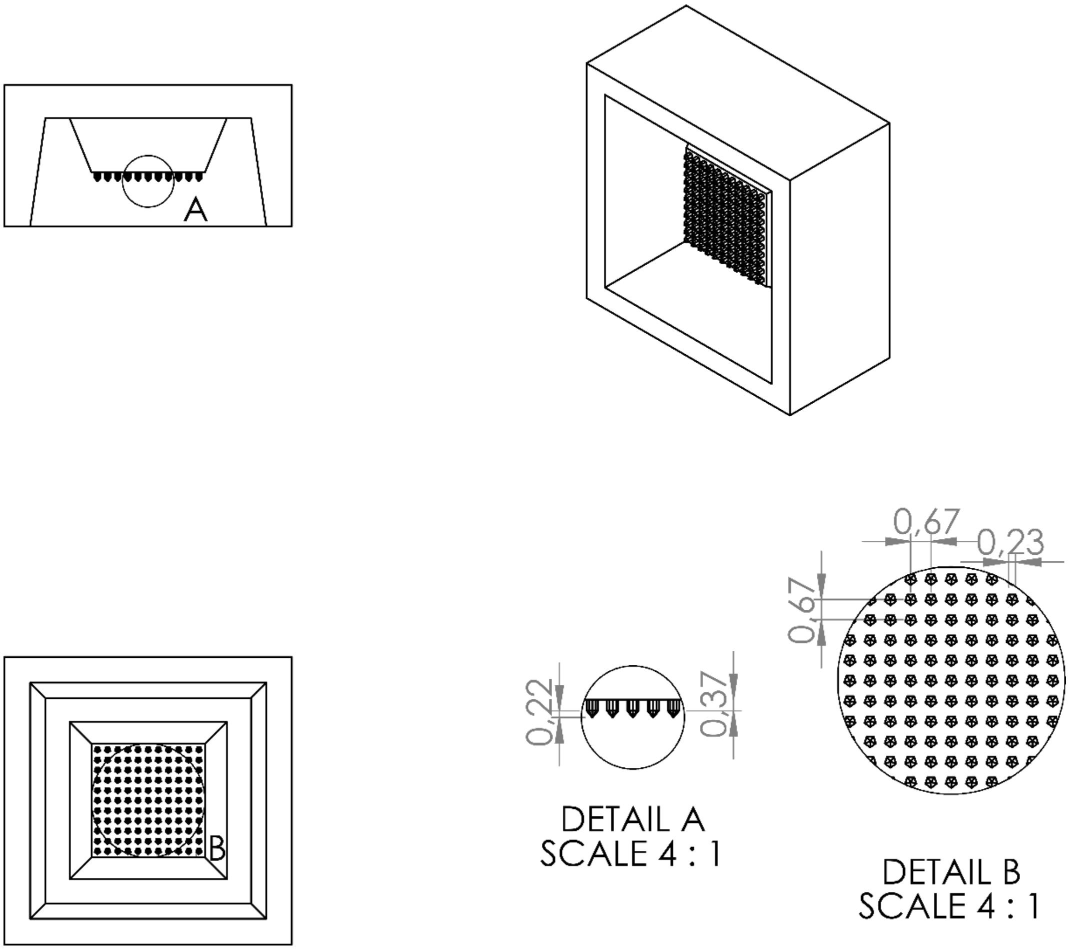

Figure S6. Technical drawings of 3D printed templates (Template to produce array design 6).

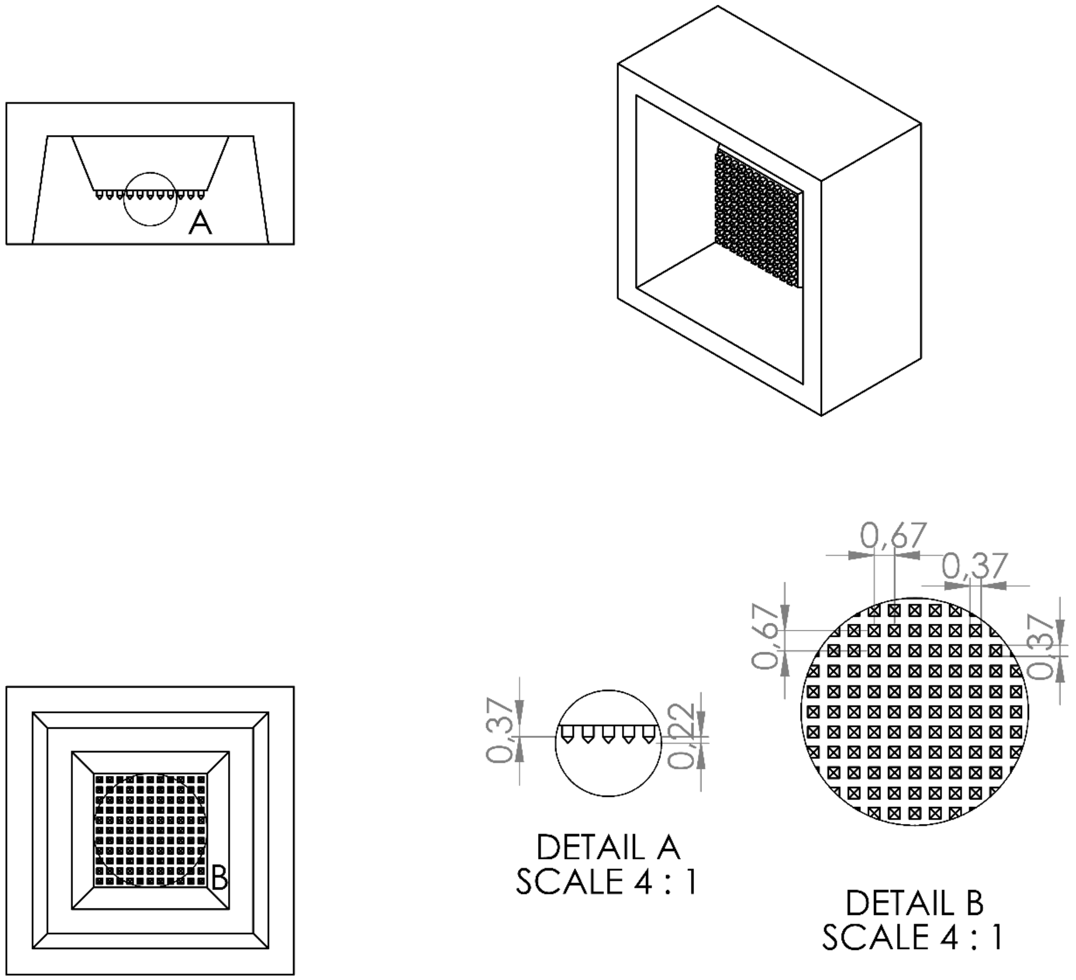

Figure S7. Technical drawings of 3D printed templates (Template to produce array design 7).

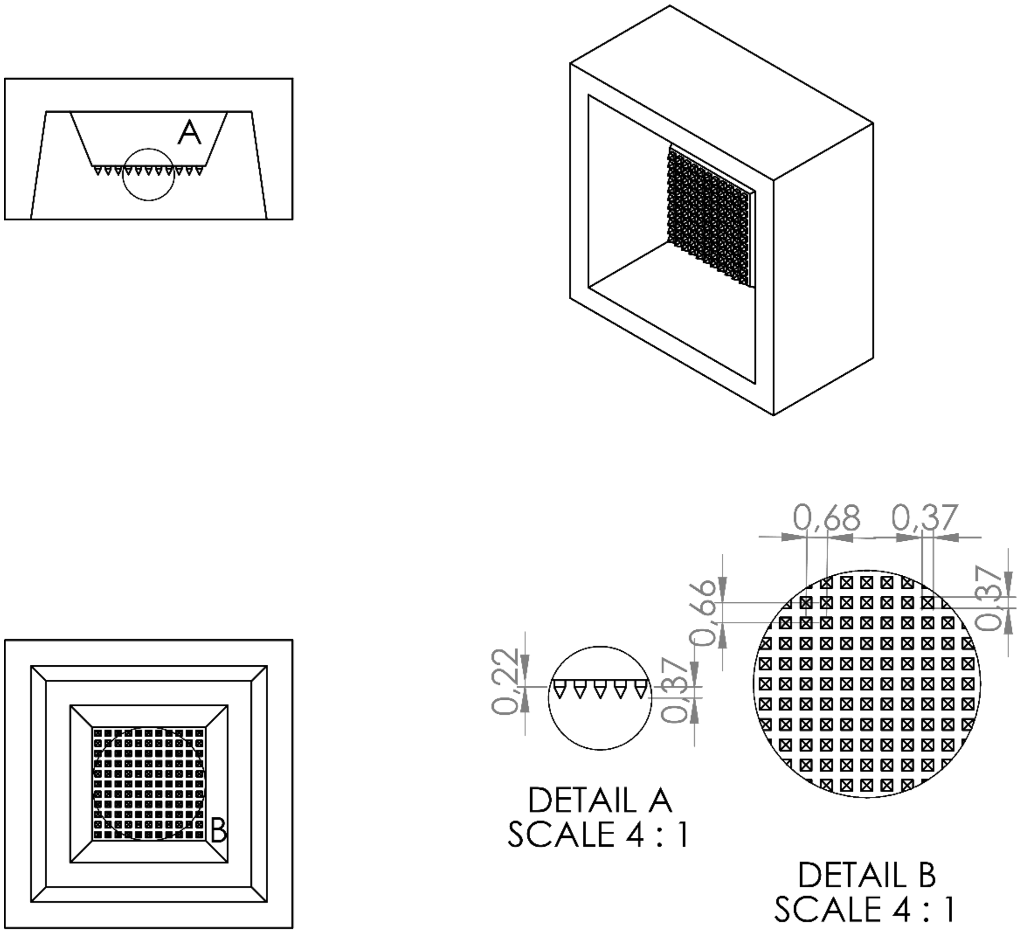

Figure S8. Technical drawings of 3D printed templates (Template to produce array design 8).

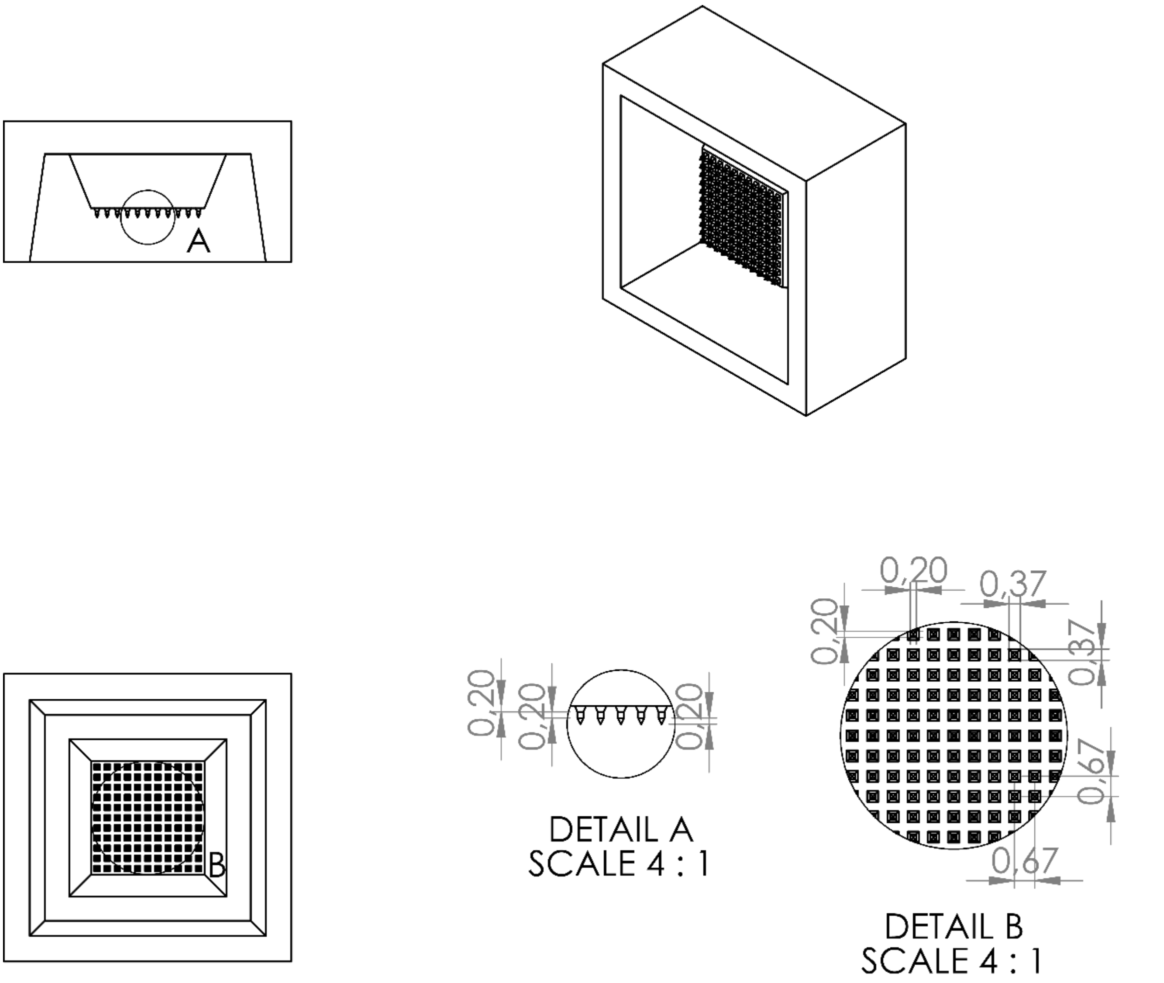

Figure S9. Technical drawings of 3D printed templates (Template to produce array design 9).

Table S2. Hydrogel formulations.

Formulation	Composition
A	PEGDMA (550 Da) 20 mL, EGDMA 4 mL, BPO 0.165 g.
B	PEGDMA (550 Da) 20 mL, EGDMA 8 mL, BPO 0.165 g.
C	PEGDMA (550 Da) 20 mL, EGDMA 2 mL, BPO 0.165 g.
D	PEGDMA (550 Da) 10 mL, EGDMA 3 mL, BPO 0.083 g.
E	PEGDMA (550 Da) 10 mL, EGDMA 5 mL, BPO 0.083 g.
F	PEGDMA (550 Da) 0.5 mL, EGDMA 0.1 mL, BPO 0.005 g, PEGDMA (20 kDa) 0.102 g, DMSO 0.1 mL.
G	PEGDMA (550 Da) 10 mL, EGDMA 2 mL, BPO 0.010 g, DMSO 2 mL.

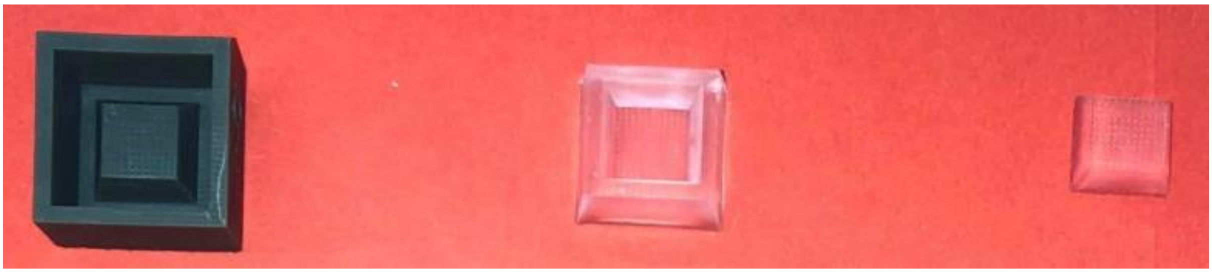

Figure S10. Photograph of exemplar structures. Left to right) 3D printed template, PDMS mold, and hydrogel microneedle array.

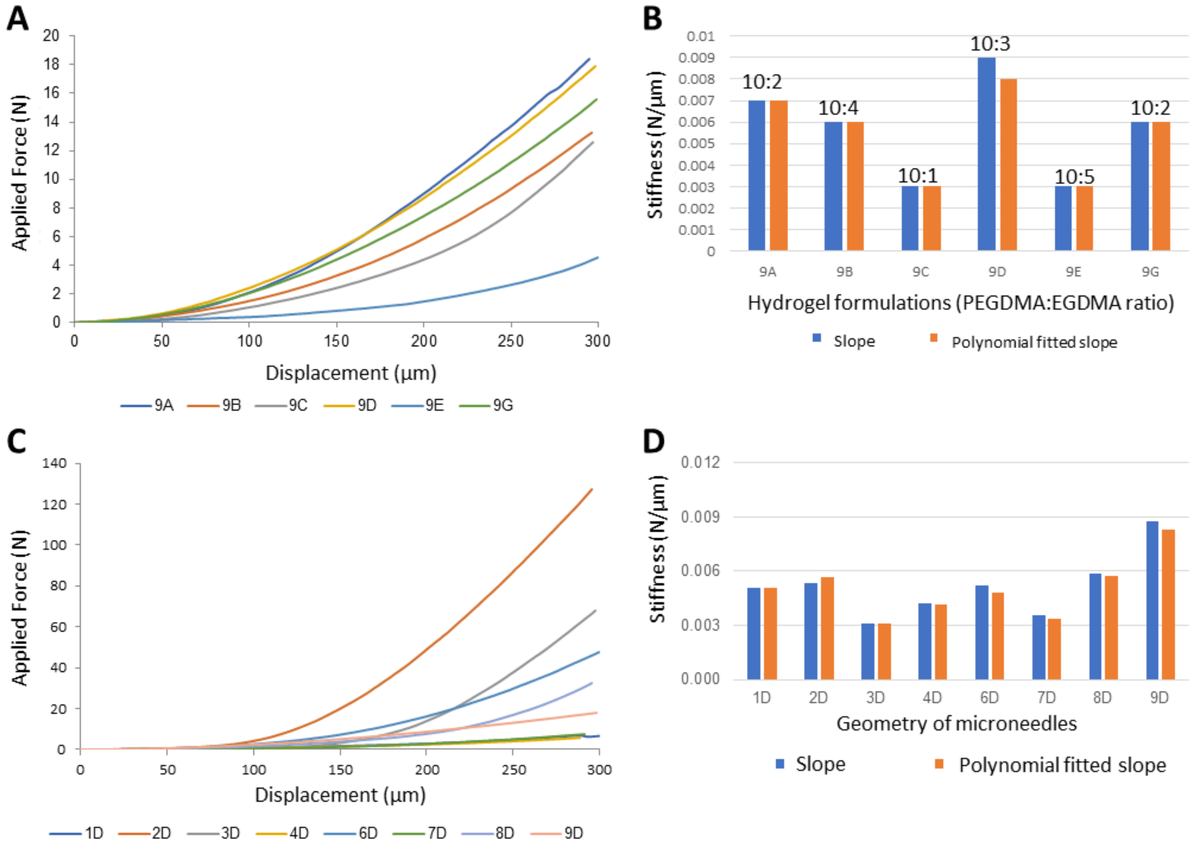

Figure S11. Mechanical tests on hydrogel microneedles based on different microneedle designs (Table S1) and hydrogel formulations (Table S2). **A)** Force-displacement curves obtained from compression test for triangle pyramid PEGDMA-based hydrogel microneedles. **B)** Effect of crosslinking ratio on rigidity. **C)** Force-displacement curves obtained from compression test for PEGDMA-based hydrogel microneedles with different geometries. **D)** Effect of microneedle geometry on rigidity of PEGDMA-based hydrogel microneedles.

Table S3. Dimensions of the moulds and hydrogels.

	Height [μm]	Apex angle [°]	Length of side at base [μm]	Interior angle of face at base [°]	Length of side at tip [μm]
Nominal mould dimensions (from CAD)	590	30.1	370	60	0
Printed mould dimensions (measured)	343.2 ± 44.7	37.6 ± 1.3	387.2 ± 24.4	60.3 ± 2.2	7.5 ± 4.5
PDMS template dimensions (measured)	316 ± 38	42.2 ± 0.68	386.3 ± 23.1	60.6 ± 3.1	0
pHEMA Microneedles dimensions (measured)	238 ± 97	43.1 ± 0.42	386.9 ± 23.2	61.1 ± 3.3	0

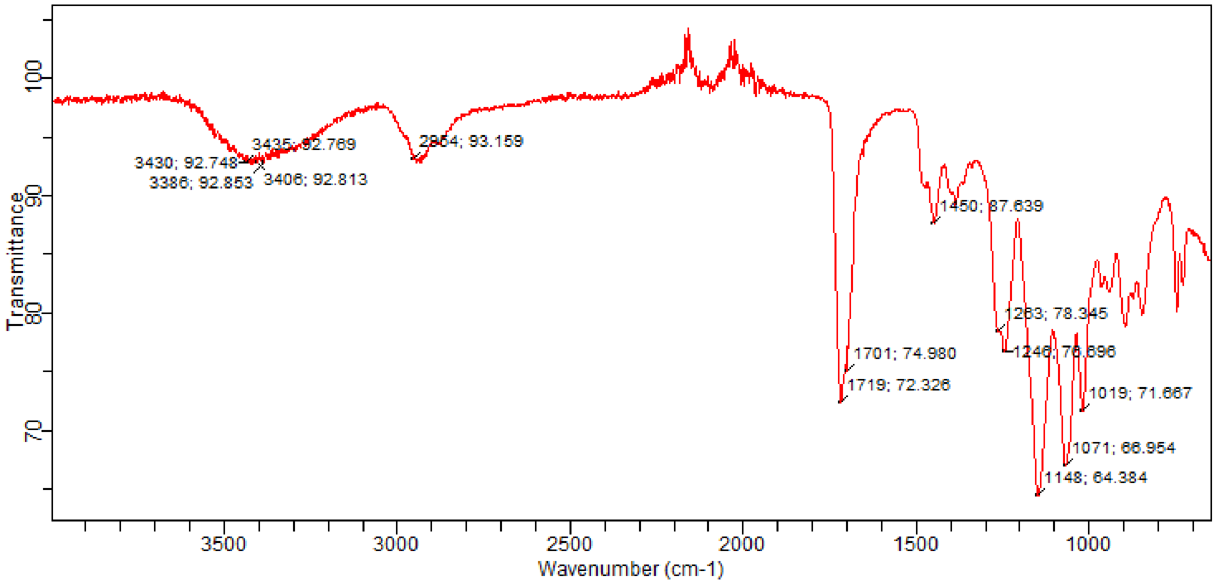

Figure S12. FTIR spectrum of hydrogel microneedles.

Hydrogel swelling studies: Gel samples were dried in a vacuum oven at 60 °C overnight. The mass of the dried gels was recorded and then transferred to a container of phosphate buffered saline (PBS) at a specific temperature (i.e., 20 °C, 35 °C, 60 °C) to study the % swelling using the equation:

$$S = \frac{(M_s - M_d)}{M_d}$$

Where S is the % swelling, M_s is weight of the swollen hydrogel at time t, and M_d is the weight of the original dry hydrogel. [1]

Swelling rate was estimated by measurement of gel mass at different time points, where M_t is the swelling content at any time (g/g d.b.) and $M_{(t+\Delta t)}$ represents the swelling content based on the dry content at t+Δt. [2]

$$W_R = \frac{M_{t+\triangle t} - M_t}{\triangle t}$$

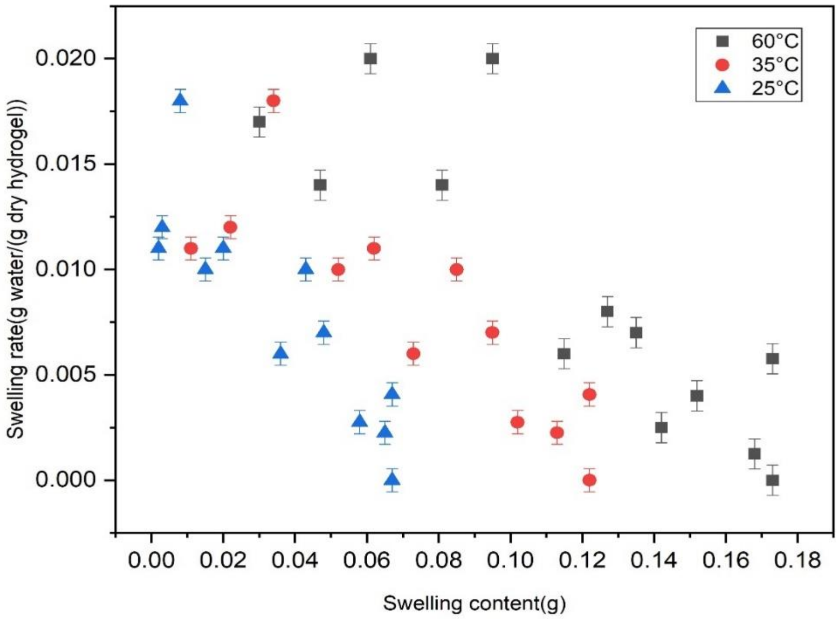

Figure S13. Swelling rate of hydrogels at different temperatures.

The swelling behavior of the hydrogels was fitted to the following equation:

$$F = \frac{M_t}{M_e} = kt^n$$

Where, F is the swelling fraction, M_t and M_e are the amounts of water diffused into the hydrogels at time t, and at equilibrium (e), respectively; k is a kinetic constant and n is the swelling exponent which describes the type of water diffusion in line with **Table S4**.

Table S4. Water diffusion in the hydrogels.

Swelling exponent (n)	Mechanism
< 0.5	Fickian diffusion (polymer chains have high mobility).
0.5 < n < 1	Non-Fickian diffusion, where anomalous transport (coupling Fickian diffusion and polymer relaxation/degradation) is observed.
1	Case 2 diffusion (time independent).
> 1	Non-Fickian super case 2.

The diffusion coefficient can be calculated using the following equation:

$$D = \pi r^2 (\mathrm{k}/4)^{1/n}$$

Where "D" represents the coefficient of diffusion as "$m^2\ s^{-1}$" and "r" represents the swollen gels radius. The mechanism of water diffusion in the hydrogels was determined from plots of ln$F$ versus ln$t$ (**Figure S14**), and the values of n, k and regression coefficients were calculated from the slopes and the intercepts (**Table 1**). The calculated swelling exponent values (n) are between 0.40 and 0.58, displaying Non-Fickian character at 20 °C and Fickian character at 35 °C, and 60 °C (**Table 1**). The diffusion coefficients (D) for the hydrogels were observed to increase with increasing temperatures (D of 0.8, 1.4, and 1.9 x 10^7 $m^2\ s^{-1}$ at 25 °C, 35 °C, and 60 °C, respectively). [2]

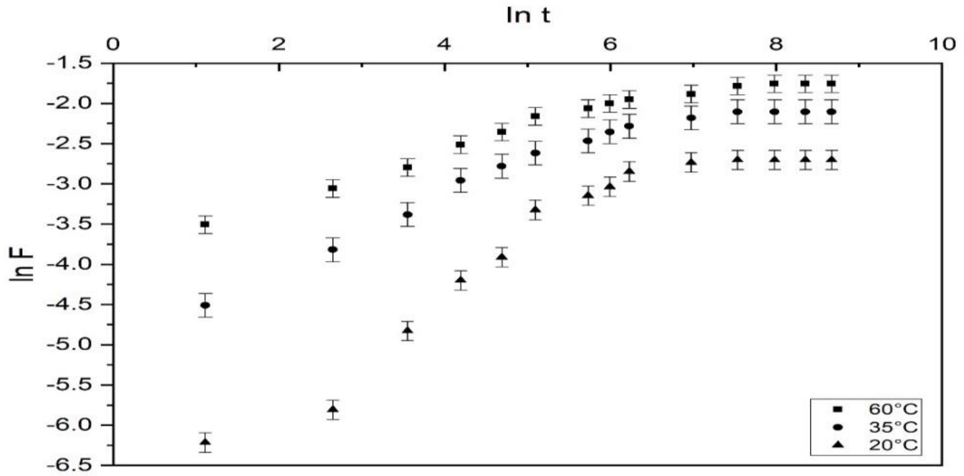

Figure S14. Swelling kinetic curves for hydrogel at different temperatures, ln*F* against ln*t*.

The Arrhenius plot (**Figure S15**) was obtained by plotting the logarithm of the rate constant, *k*, versus the inverse temperature, *1/T*. The resulting negatively-sloped line (a straight line with R^2 of 0.99) facilitates finding the missing components of the Arrhenius equation. [3]

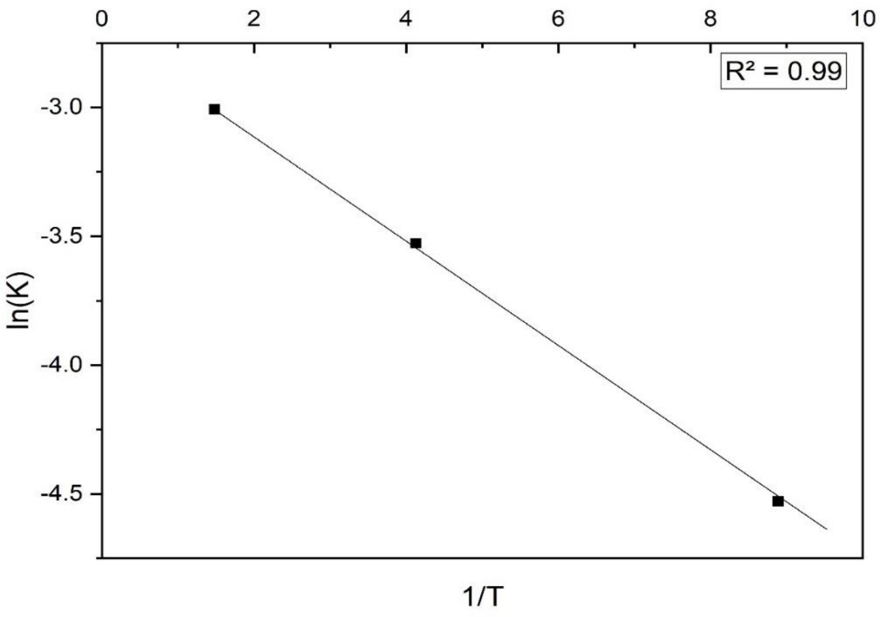

Figure S15. Arrhenius plot: the logarithm of the rate constant, *K*, versus the inverse temperature, *1/T*.

Extrapolation of the line back to the y-intercept yields the value for ln A. The slope of the line is equal to the negative activation energy divided by the gas constant, R. The activation energy (*Ea*) is derived from the Arrhenius equation.

$$K = Ae^{-Ea/_{RT}}$$

Where, A represents the pre-exponent which is a constant, E_a is activation energy, R is the universal gas constant of 8.314×10^{-3} (kJ mol^{-1}K^{-1}), K is the rate constant. From the plot A_0 (pre-exponential factor of Arrhenius equation) activation energy was calculated. The activation energy (*Ea*) was found to be 29.88 KJ mol^{-1}, and the pre-exponential factor (A_0) of the Arrhenius equation or frequency factor was found to be 2.66×10^{-3} s^{-1}.

$$D = A_o \exp((-E_a)/(R(T+273.15)))$$

Where A_0 is the preexponential factor of Arrhenius equation (m^{2}s^{-1}), E_a is the activation energy (kJ mol^{-1}), T is temperature of water (K^{-1}), and R is the universal gas constant of 8.314×10^{-3} (kJ mol^{-1}K^{-1}). The graph for Arrhenius-type relationship between effective diffusivity coefficient and temperature (In(D) vs (1/K)), indicates Arrhenius dependence. [2]

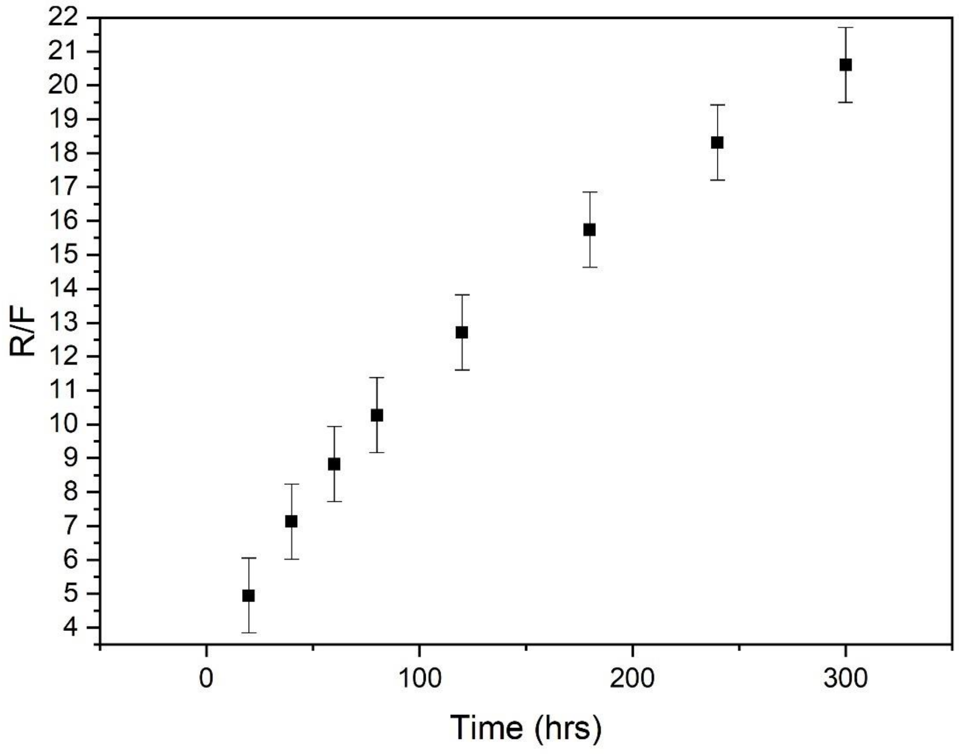

Figure S16. Relaxation of the polymer chains.

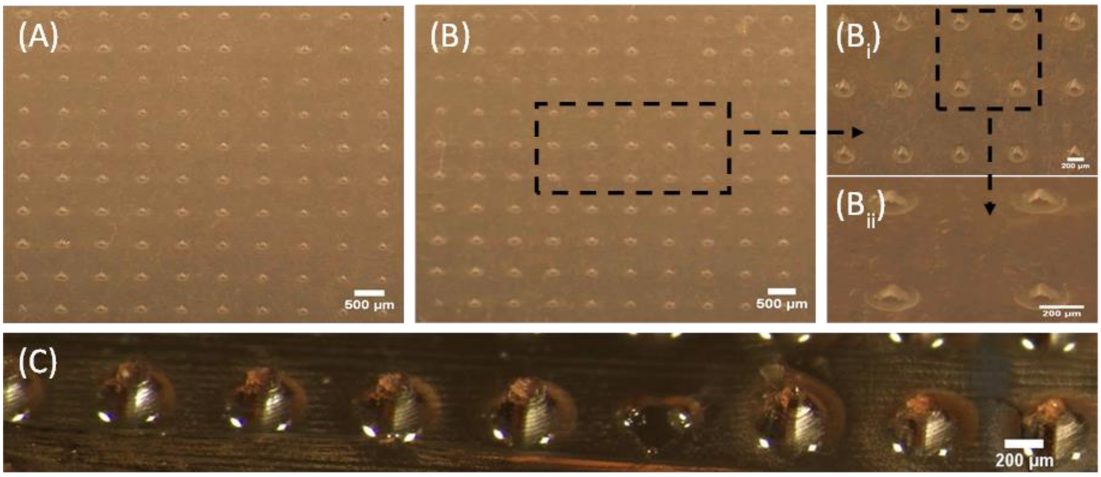

Figure S17. Optical images of Parafilm ® multilayer film skin mimic after (A) first penetration with 10N force, (B) 20th penetration with 10N force, and (C) appearance of the microneedles after penetration tests.

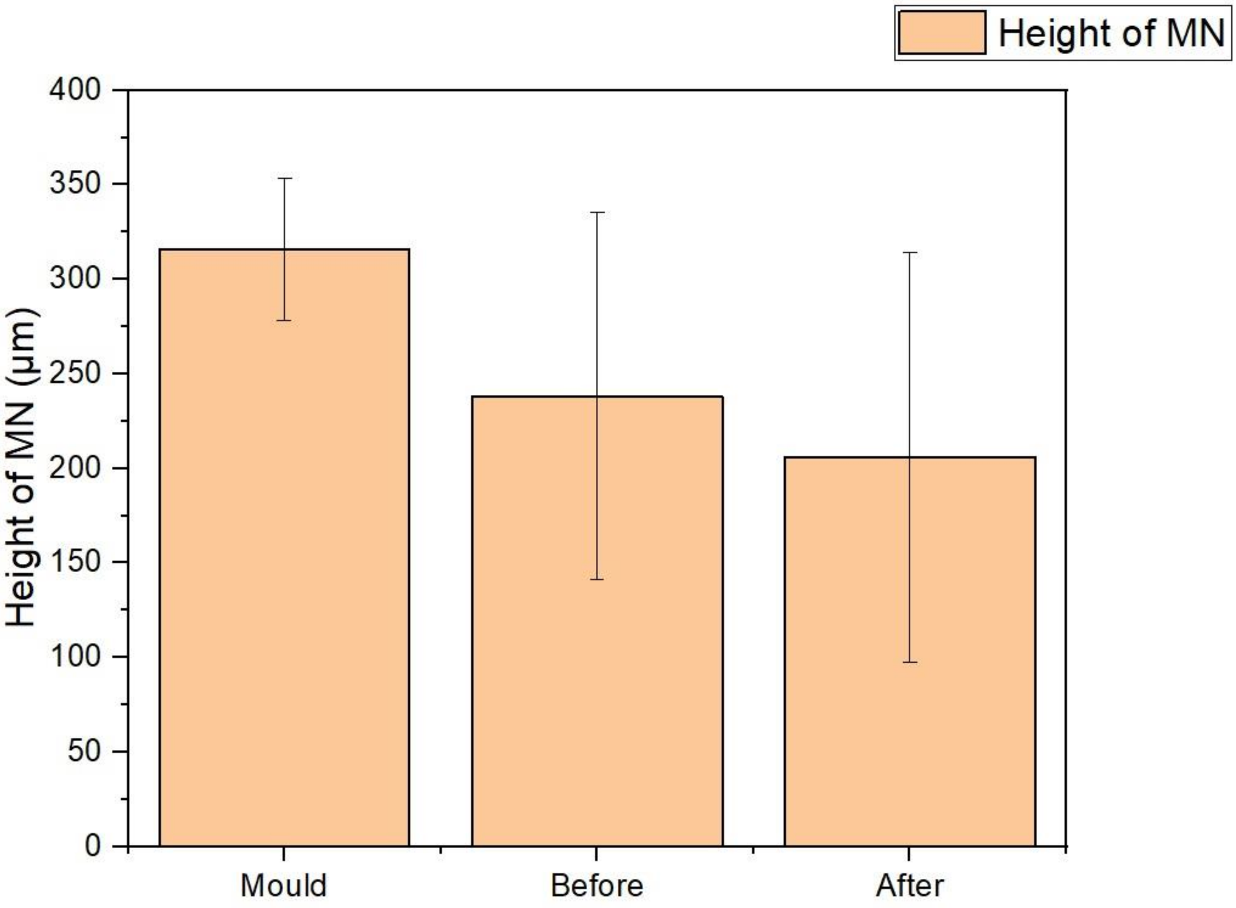

Figure S18. Microneedle array heights before and after penetration of multilayer films of Parafilm ®. Microneedles inserted with a force of 40 N.

Drug delivery experiments: UV-vis spectroscopy was used to assess the concentration of metformin in solution in PBS at pH 7.4, at a wavelength of 625 nm at various times, and correlated to a calibration curve to enable study of cumulative release using the Beer–Lambert law ($A = \varepsilon lc$), where A is absorbance, ε is the molar extinction coefficient (which depends on the nature of the chemical and the wavelength of the light used), l is the length of the path light must travel in the solution in centimetres, and c is the concentration of a given solution. All the experiments were in triplicate (n = 3), and data is reported as the mean average ± standard deviation. UV-vis calibration curves for metformin absorption (with an R^2 value of 1) were used for least squares linear regression analysis and correlation analysis. The limit of detection (LoD) was calculated from:

$$LoD = (3.3 \times \sigma) \div S$$

Where, S is Slope, Pi is the SD of intercept; the LoD was found to be 36.8 ppm. The limit of quantification (LoQ) was calculated from:

$$LoQ = (10 \times \sigma) \div S$$

The LoQ was found to be 111.4 ppm.

Zero order model release kinetics: Zero order release kinetics describe systems where the drug release rate is constant over a period of time. The equation for zero order release is:

$$[C] = -K_t = [C]_0$$

$$C_0\text{-}C_t = K_{0t}$$

$$C_t = C_0 + K_{0t}$$

Where, C_t is the amount of drug released at time t, C_0 is the initial concentration of drug at time t = 0, K_0 is the zero order rate constant. Thus, zero order kinetics defines the process of constant drug release from a drug delivery system and drug level in the medium remains constant throughout the delivery.[5]

First order model release kinetics: The equation for first order release is:

$$\ln[C] = -k_t + lnC_0$$

After rearranging and integrating the equation,

$$log\ C = log\ C_0\text{-}K_0^{t/2.303}$$

K_1 is the first order rate equation expressed in t^{-1} or per hour, C_0 is the initial concentration of the drug, C is the percentage of drug remaining at time t. Hence, log % of drug remaining vs.

time and the slope of the plot gives the first order rate constant. The correlation coefficient of the above plot will give the information whether the drug release follows first order kinetics or not.[5]

Second order model release kinetics: The equation for second order release is: [6]

$$\frac{1}{[C]} = k_t + [\frac{1}{C_0}]$$

References

1. Gupta, N.V. and H.G. Shivakumar, *Investigation of Swelling Behavior and Mechanical Properties of a pH-Sensitive Superporous Hydrogel Composite.* Iran J Pharm Res, 2012. **11**(2): p. 481-93.
2. Kipcak, A.S., et al., *Modeling and Investigation of the Swelling Kinetics of Acrylamide-Sodium Acrylate Hydrogel.* Journal of Chemistry, 2014. **2014**: p. 281063.
3. Weng, L., X. Chen, and W. Chen, *Rheological Characterization of in Situ Crosslinkable Hydrogels Formulated from Oxidized Dextran and N-Carboxyethyl Chitosan.* Biomacromolecules, 2007. **8**(4): p. 1109-1115.
4. Waghule, T., et al., *Microneedles: A smart approach and increasing potential for transdermal drug delivery system.* Biomedicine & Pharmacotherapy, 2019. **109**: p. 1249-1258.
5. *5 - Mathematical models of drug release*, in *Strategies to Modify the Drug Release from Pharmaceutical Systems*, M.L. Bruschi, Editor. 2015, Woodhead Publishing. p. 63-86.
6. Bagshaw, C.R., *Order of Reaction*, in *Encyclopedia of Biophysics*, G.C.K. Roberts, Editor. 2013, Springer Berlin Heidelberg: Berlin, Heidelberg. p. 1807-1808.
