# Supplementary material for: Poly(2‐Hydroxyethyl Methacrylate) Hydrogel‐Based Microneedles for Metformin Release
Source: Glob Chall. 2023 Jul 5;7(8):2300002. doi: 10.1002/gch2.202300002 (PMC10448145; doi:10.1002/gch2.202300002)
Supplement: Supplementary file 2 — Supporting Information [file GCH2-7-2300002-s001.zip › gch2202300002-sup-0002-SuppMat/WGWP13469_ACR.pdf]

ACR Sheet

Editorial Ref. ID	gch2.202300002
WJPCMS No.	gch2202300002
DOI	10.1002/gch2.202300002
Article Title	Poly(2-hydroxyethyl methacrylate) hydrogel-based microneedles for metformin release
Corresponding Author	Aljohani, J. G. Hardy
Email	j.g.hardy@lancaster.ac.uk
AA Article?	No
NIH Funded?	No
Authors names for NIH	
Supporting Material Available?	Yes
Supporting Material Detail	SuppMat
Received Date	11/01/2023 16:00
Revised Date	21/05/2023 16:00
Accepted Date	24/05/2023 16:00
WB Received Date	24/05/2023 16:00
CTA Type	CTA - WALS awaited
CTA/ELF Date	
Online Open	No
Article Type	Research Article
ToC Category	
Graphical ToC	Yes
Graphical Abstract	No
Bookingid	WGWP13469
PA Comments	
PM Comments	
AID	
Society No.	GCH21519
Batch No.	0
Acronym	No
MSP Pages	22
Keywords	Yes
Summary	Yes
Reference List	Yes
Missing Materials	0
Key Boarding	0
No. of Tables	1
No. of Figures	4
Color Paid Figure(s) #	0
Color Gratis Figure(s) #	0
CoW Figure(s) #	1
Associate editor	0
Subject Code	[121] Editorial
Conflict of Interest	No
Source Type	docx
Service Type	Full Service
Category	NA
AID	
Embargo	N
Embargo Date	
Fund Selection	
Legal Statement	

Copyrightline	© ([0-9]+) The Authors. Global Challenges published by Wiley‐VCH GmbH
